# Indium Doping of Lead-Free Perovskite Cs_2_SnI_6_

**DOI:** 10.3389/fchem.2020.00564

**Published:** 2020-08-04

**Authors:** Shodruz T. Umedov, Anastasia V. Grigorieva, Leonid S. Lepnev, Alexander V. Knotko, Koji Nakabayashi, Shin-ichi Ohkoshi, Andrei V. Shevelkov

**Affiliations:** ^1^Department of Materials Science, Lomonosov Moscow State University, Moscow, Russia; ^2^Department of Chemistry, Lomonosov Moscow State University, Moscow, Russia; ^3^Lebedev Physical Institute of the Russian Academy of Sciences, Moscow, Russia; ^4^Department of Chemistry, School of Sciences, University of Tokyo, Tokyo, Japan

**Keywords:** perovskite photovoltaics, cesium iodostannate(IV), solid solution, Raman spectroscopy, optical characteristics

## Abstract

Structure and properties of an inorganic perovskite Cs_2_SnI_6_ demonstrated its potential as a light-harvester or electron-hole transport material; however, its optoelectronic properties are poorer than those of lead-based perovskites. Here, we report the way of light tuning of absorption and transport properties of cesium iodostannate(IV) Cs_2_SnI_6_ via partial heterovalent substitution of tin for indium. Light absorption and optical bandgaps of materials have been investigated by UV-vis absorption and photoluminescent spectroscopies. Low-temperature electron paramagnetic resonance spectroscopy was used to study the kind of paramagnetic centers in materials.

## Introduction

The efficiency of third-generation solar cells (SCs) based on materials with a perovskite structure is growing every year (Elumalai et al., 2016; Li et al., 2016; Powalla et al., 2018; Sani et al., 2018; Ajay et al., 2019). Such rapid success of using these materials in various fields of solar photovoltaics is due to their unique optoelectronic properties. Today, lead-based perovskite materials (APbX_3_) are the most efficient in terms of converting solar radiation into electricity (Brandt et al., 2015; Dimesso et al., 2017; Yang et al., 2017; Zhu Z. et al., 2019), and it is caused by low binding energy of excitons, charge carrier mobility, long diffusion length, high absorption coefficient, and direct bandgap (Xiao and Yan, 2017; Deng et al., 2019). The theoretically calculated highest power conversion efficiency (PCE) (Shockley–Queisser limit) achieved by lead-based perovskite is 31.4% for CH_3_NH_3_PbI_3_ (Wan-Jian et al., 2015) and the experimental efficiency (light converting efficiency) of SCs with this compound has exceeded 25% (Yang et al., 2017; Jeon et al., 2018; Powalla et al., 2018). However, the chemical and thermal stability of lead perovskites are not sufficiently well; moreover, lead is a toxic element (Kulbak et al., 2016; Wu Y. et al., 2017; Pisanu et al., 2019). These problems motivate finding new alternatives among lead-free materials with optimal optoelectronic characteristics (Hoefler et al., 2017; Fu, 2019; Pisanu et al., 2019). Instead of lead perovskites, materials with general formulas of ABX_3_ [where A = CH_3_${\text{NH}}_{3}^{+}$, ${\text{HC}({\text{NH}}_{2})}_{2}^{+}$, or Cs^+^, Rb^+^, K^+^; B = divalent inorganic cation; X = Cl, Br, I] and A_2_BX_6_ (where A and X the same cations and anions as in the case of ABX_3_; B = tetravalent inorganic cations) are in focus for investigations (Huang and Lambrecht, 2013; Stoumpos et al., 2016; Cai et al., 2017; Ju et al., 2017). Among all lead-free compounds with the ABX_3_ formula, cesium triiodostannate CsSnI_3_ turned out to be the most promising material with good optoelectronic performance (Kumar et al., 2014, 2017; da Silva et al., 2015; Stoumpos et al., 2016). The first SCs based on Sn^2+^ perovskites had a very low efficiency (3 × 10^−4^%−2%) (Chen et al., 2012; Kumar et al., 2014). Nevertheless, CsSnI_3_ has excellent properties [optimal bandgap of 1.4 eV, higher theoretical short-circuit photocurrent (*J*_*SC*_) density of 34.3 mA cm^−2^, highest hole mobility ~585 cm^2^ V^−1^ s^−1^ among p-type materials] for photovoltaic application as light harvesting or p-type semiconductor (Chung et al., 2012; Stoumpos et al., 2013). Thereby, the CsSnI_3_ phase is still being actively studied (Song et al., 2018; Wijesekara et al., 2018; Pisanu et al., 2019) and the PCE of SCs based on it has achieved 5.03% (Wang Y. et al., 2019), but these results are still less satisfactory than those of MAPbI_3_.

Another type of compounds are double perovskites, which are obtained by replacing the tetravalent cation B^4+^ and a vacancy in A_2_BX_6_ with the B^+^/B^3+^ pair (where B^+^ = Cu, Ag, Ga, In, Tl, etc.; B^3+^ = Sb, Bi etc.) so that the charge neutrality is preserved (Yin et al., 2019). According to Savory et al. (2016), the theoretical calculations show a PCE limit <8% for the double perovskite compound Cs_2_AgBiX_6_; however, higher PCE and suitable bandgaps have been predicted for other materials such as Cs_2_InSbCl_6_, Cs_2_AgInBr_6_, Rb_2_AgInBr_6_, and Rb_2_CuInCl_6_ (Zhao et al., 2017a,b). According to Xiao et al. (2019), the most promising double perovskites are based on cations such as Ag, In, Bi, and Sb (Greul et al., 2017; Wu C. et al., 2017; Gao et al., 2018; Liang and Gao, 2018; Fan et al., 2019; Igbari et al., 2019).

A promising lead-free non-toxic and stable perovskite-like material with a face-centered cubic cell is A_2_SnX_6_, where the tin atom is strongly covalently bonded and stabilizes the crystal lattice. Among all compounds in the A_2_SnX_6_ group, the Cs_2_SnI_6_ phase turned out to be a suitable material for photovoltaics (Cai et al., 2017; Maughan et al., 2018). Cesium iodostannate(IV) has promising optoelectronic properties (electron mobility up to 509 cm^2^ V^−1^ s^−1^; Guo et al., 2017; *J*_*SC*_ = 13.97 mA cm^−2^; *V*_OC_ = 0.58 V; *E*_g_ = ~1.2 eV) (Kaltzoglou et al., 2016), which makes it a candidate as a light-harvesting material in SCs. Mixed cations A^+^((A_*x*_A_1−x_)_2_BX_6_) (Ganesan et al., 2019) and anions X^−^(A_2_B(X'_*x*_X_1−x_)_6_) (Lee et al., 2017; Ke et al., 2018; Yuan et al., 2019; Zhu W. et al., 2019) were considered to improve stability and properties of Cs_2_SnX_6_. Upon replacement of Cl^−^ by I^−^, the optical absorption coefficient increases, the bandgap decreases, stability decreases, and the materials color changes from pale yellow (or white) to black. The Cs_2_SnI_6_ phase is also used as photodetectors (Han et al., 2019), for photoelectrochemical water splitting (Dang et al., 2019), as photocatalysts (Wang X.-D. et al., 2019), and the efficiency of the SCs based on it has reached ~11.2%. In this work, we have demonstrated the preparation of solid solutions based on Cs_2_SnI_6_ by doping with indium. Heterovalent substitution of tin (Sn^4+^ ionic radii 0.69 Å, Pauling electronegativity = 1.96) in the structure of Cs_2_SnI_6_ by indium (In^3+^ ionic radii 0.74 Å, Pauling electronegativity = 1.78) leads to the formation of solid solutions with improved optical properties. Here, for the In^3+^ → Sn^4+^ substitution, the difference in electronegativities of cations is 0.18 and the difference in ion sizes is 7.24%, which corresponds well to the Goldschmidt rule (Goldschmidt, 1926).

Recently, the new wave of enthusiasm among researchers has arrived from coming perspectives of nanocrystalline Cs_2_SnI_6_ phase and its derivatives for optoelectronics (Wang et al., 2016; Veronese et al., 2020). Because of this, development of nanochemistry of complex halides requires preliminary fundamental studies of phase equilibrium and analysis of optical and transport characteristics in polycrystalline materials.

## Experimental Section

### Syntheses of Materials

The sintering was carried out by solid-state sintering method in evacuated quartz ampules (RT pressure of 1.6 · 10^−2^ Torr). The compositions were prepared by grinding cesium iodide CsI (Sigma-Aldrich 99.99%), tin iodide SnI_4_ [direct synthesis from elementary tin (“Ruschim,” 99.90+, O-1) and iodine in CCl_4_ (purum, “Irea2000”) with further purification by sublimation at 270°C], and elementary iodine (purum, “Reachim”) and metallic indium (“Ruschim,” 99.999%) with the stoichiometric mass ratios. The pristine Cs_2_SnI_6_ phase was obtained with the stoichiometric mass of CsI and SnI_4_ (2:1). The mixtures were sealed in preliminarily dried quartz ampoules and heated with the rate of ~0.2°C/min to 300°C and then annealed at this temperature for 48 h. All samples were kept in double closed zip-lock bags in nitrogen.

All materials were characterized with powder XRD method for phase composition definition. We assumed that the obtaining materials have the composition related to the solid solutions Cs_2−x_Sn_1−x_In_*x*_I_6−2x_ based on Cs_2_SnI_6_ or CsInI_4_ phases. The following chemical equation shows the phase composition and yield of the reaction products.

(1) $$\begin{array}{r} \left(2-x\right)\text{CsI}+\left(1-x\right)\text{Sn}{\text{I}}_{4}+\left(x\right)\text{In}+\left(1.5x\right){\text{I}}_{2}\to \\ {\text{Cs}}_{2-x}{\text{Sn}}_{1-x}{\text{In}}_{x}I_{6-2x} \end{array}$$

### Characterization Methods

The ampoules with samples were open right before the following analyses. The samples were transferred to the closely packed cells for further storing. XRD, Raman, and UV-vis measurements were performed in air for 15 min.

X-ray diffraction measurements (XRD) were performed on a Rigaku D/MAX 2500 diffractometer equipped with a rotating copper anode (Cu-Kα radiation) and operated at 45 kV and 250 mA from 5° to 80° in 2Θ; at the continuous scanning speed 5° min^−1^ with a measuring step of 0.02°. The experimental data were analyzed using WinXPow (database PDF2) to define the phase composition, and Jana2006 software was used for unit cell parameter calculations.

To analyze the optical properties, the samples were studied by diffuse reflection spectroscopy. One gram of each compound was placed in the cell of the spectrophotometer and pressed tightly with a quartz glass and then measured in the range of 1,400–200 nm with a scan step of 1 nm. UV-vis diffuse reflectance spectra were collected using a UV-vis spectrometer Lambda 950 (PerkinElmer). All measurements were performed at 298 K with a scanning rate of 2 nm/s using quartz glass as a reference. Reflectance (*R*) was converted to absorption (α) data in accordance with the Kubelka–Munk model: α/*S* = (1 – *R*)^2^/(2·*R*). The optical energy bandgap (*E*_g_) was acquired using a Tauc plot, the dependence of (αhν)^2^ on energy (hν).

Emission spectra were collected with a multichannel spectrometer S2000 (Ocean Optics) with a nitrogen LGI-21 (λ_ex_ = 337 nm) as an excitation source at 293 K and 77 K. All spectra were corrected for the wavelength response of the system. Additionally, the photoluminescence emission spectra of the sample CsInI_4_ was investigated with a diode source of 365 nm.

EPR spectra were recorded using a X-band JES-FA200 (JEOL) spectrometer at the temperature of 294–4.2 K. The modulation frequency is 100 kHz and microwave frequencies are around 9.00 GHz. The samples are put in a quartz tube with an upper glass part, then purged by Ar gas and vacuum three times, and finally sealed in vacuum (about 20 Pa).

## Results and Discussion

**Figure 1:**
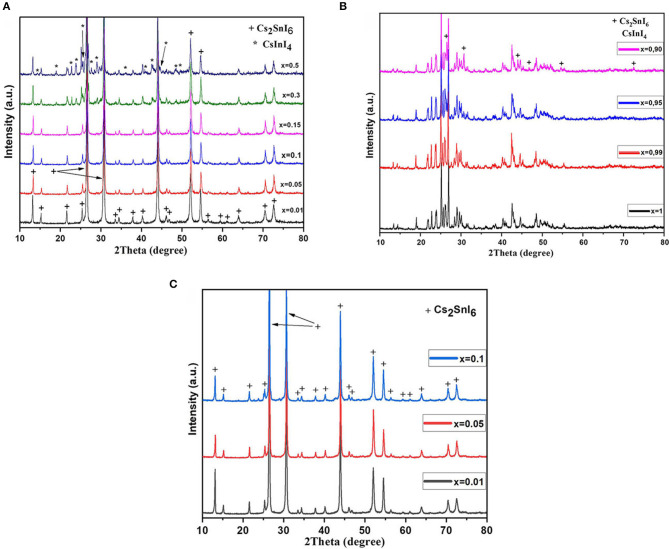
**(A)** Powder XRD data for Cs_2−x_Sn_1−x_In_*x*_I_6−2x_ (*x* = 0.01–0.1) compounds. **(B)** XRD data of Cs_2−x_Sn_1−x_In_*x*_I_6−2x_ with high substitution rate *x* (*x* = 0.9 – 1). All unmarked reflections belong to the CsInI_4_ phase. **(C)** XRD data of three samples with *x* = 0.01, 0.05, 0.1 ratio after a year of storage in air. “+” denotes Cs_2_SnI_6_ reflections, and “*” indicates reflections of CsInI_4_.

As shown in Figure 1A, about 10% of tin atoms in the structure of Cs_2_SnI_6_ can be substituted by indium atoms. All reflections on the corresponding XRD patterns belong to the Cs_2_SnI_6_ with a cubic structure and space group F*m-3m*(225) (PDF2 file #73-330). Diffraction patterns for the compositions with *x* from 0 to 0.15 display a shift of the reflections toward lower 2θ upon increasing the substitution rate. The increase of the unit cell parameter *a* compared to pure Cs_2_SnI_6_ phase indicates a slight increase of the unit cell volume. According to the fact that the ionic radius of the In^3+^ (0.74 Å) is slightly larger than that of Sn^4+^ (0.69 Å), the cell volume increases. At higher substitution rates, *x* of 0.3 and 0.5, reflections of the CsInI_4_ phase (PDF2 file #76-2101) with a monoclinic structure [P*21/c* (14) space group] are observed on diffraction patterns of the respective samples. The estimated cell parameters of the F-centered cubic Cs_2_SnI_6_ are given in Table 1. Such gentle change of the unit cell parameters is probably due to the formation of iodine vacancies in the anion sublattice.

**Table 1:** XRD data for Cs_2−x_Sn_1−x_In_x_I_6−2x_ compositions.

**Sample composition**	**Substitution rate *x***	**Cell parameter *a*, Å**	**Unit cell volume *V*, Å^**3**^**	***R*_**p**_**	***R*_**I**_**
Cs_1.99_Sn_0.99_In_0.01_I_5.98_	0.01	11.6505 (6)	1581.4 (1)	13.66	5.52
Cs_1.95_Sn_0.95_In_0.05_I_5.9_	0.05	11.6541 (6)	1582.8 (1)	14.34	5.80
Cs_1.9_Sn_0.9_In_0.1_I_5.8_	0.10	11.6548 (3)	1583.1 (1)	15.91	6.32

Samples (polycrystalline powders) were stored in air under laboratory conditions for 1 year and investigated by powder XRD repeatedly during this period. According to the XRD results (Figure 1), the indium-substituted samples remain single-phase with the cubic Cs_2_SnI_6_ structure, while the pristine Cs_2_SnI_6_ phase (without doping) decomposed already after a month of storage into CsI and volatile SnI_4_, which turned into SnO_2_ as a result of hydrolysis. The stability of the substituted samples is probably due to the fact that indium with iodine form stronger ionic bonds than tin; therefore, the lattice of solid solutions is more stable than the pure phase lattice. At the same time, the sample with *x* = 0.10 has very weak reflections of admixture at 18.77°, 22.65°, and 23.66° in this XRD pattern. These reflections most probably belong to the CsInI_4_ phase. For comparison, the strongest reflections of cesium iodide CsI should be at 22.87°, 32.56°, and 52.63°. The impurity is probably a result of the segregation process.

**Figure 2:**
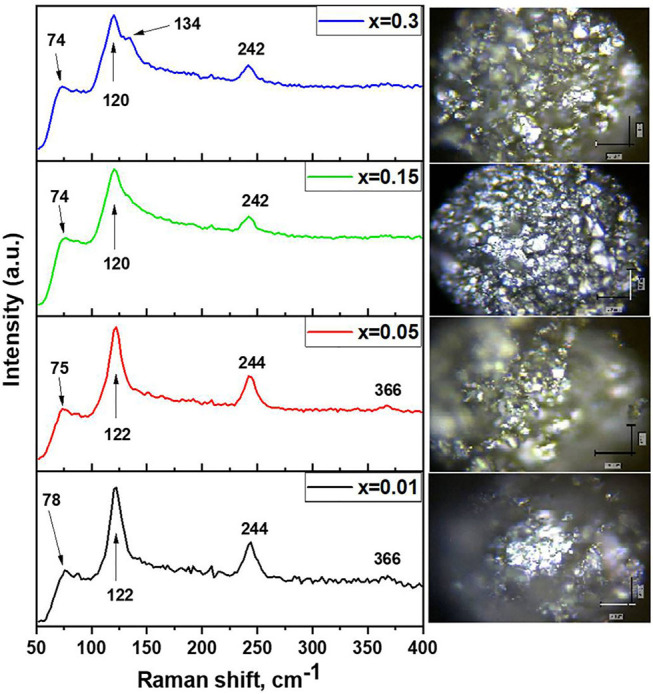
Raman spectra of samples Cs_2−x_Sn_1−x_In_*x*_I_6−2x_ (*x* = 0.01–0.3) and their optical microscopy data.

The samples of In-substituted Cs_2_SnI_6_ were studied by Raman spectroscopy. As can be seen from optical microscopy (Figure 2), the samples are homogeneous in appearance and all crystallites have a similar color. The spectra contain the strongest modes ν(A_1g_)—122 cm^−1^, ν(E_g_)—83 cm^−1^, and δ(F_2g_)—78 cm^−1^, which are related to vibrations of [SnI_6_]^2−^ octahedra [namely, ν(A_1g_) is a symmetric stretching of Sn–X bonds; δ(F_2g_) is a X–Sn–X asymmetric bending]. Substitution of Sn^4+^ with In^3+^ resulted in shortening of M–I bonds and distortion of [MI_6_]^2−^ octahedrons. Since Cs_2_SnI_6_ perfectly absorbs the visible part of the spectrum, the excitation by a green laser (wavelength, 514.4 nm) excites second- and third-order harmonics at 244 and 366 cm^−1^ due to strong resonance. The relative shift of the lines relative to the theoretical ones in the direction of smaller or larger wave numbers is associated with a changing energy and lengths of the Sn–I bonds in the [SnI_6_]^2−^ octahedra. Substitution of tin by indium results in small “left shift” and broadening of ν(A_1g_), and δ(F_2g_) Raman modes show the increase of element–iodine bond length. Raman spectra of the In-doped Cs_2_SnI_6_ differ also from the spectrum reported by Qui et al. for pure Cs_2_SnI_6_ (Qiu et al., 2017).

In the Raman spectrum of the two-phase sample *x* = 0.3, a band observed at 134 cm^−1^ corresponds to the vibration of the [InI_4_]^−^ tetrahedron. Namely, ν(A_1g_) is a symmetric stretching vibration of In–I bonds in [InI_4_]^−^ tetrahedral in CsInI_4_ phase. Other vibration modes of [InI_4_]^−^ tetrahedra do not appear in the spectra. It was found that Raman spectrum of the *x* = 0.15 sample had an additional shoulder at 134 cm^−1^. The possible reason for this is a fine and evenly distributed admixture of CsInI_4_. Its XRD reflections do not present in diffractograms, but Raman spectroscopy revealed its presence as the surface admixture. Eventually, this admixture recrystallizes, leading to larger crystallites of cesium iodogallate.

**Figure 3:**
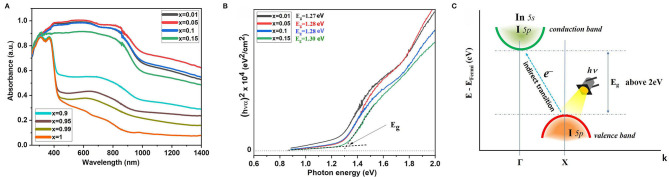
**(A)** Optical absorption spectra of the samples Cs_2−x_Sn_1−x_In_*x*_I_6−2x_ (*x* = 0.01–1.0). **(B)** Tauc plot for Cs_2_SnI_6_-based compounds, and **(C)** scheme of the electronic band structure of CsInI_4_.

The optical absorption spectra shown in Figure 3 consist of two maxima (at ~800 nm and at ~600 nm). The materials absorb from the near infrared (from 1,000 nm) to ultraviolet (UV) (380 nm). The first local maximum (~800 nm) is due to the transition of the electrons from the maximum of valence band (which is formed from I *5p* orbitals) to the minimum of conduction band formed by hybridized I *5p*–Sn *5s* orbitals. This transition is characteristic for all the samples excluding *x* = 1. The estimated bandgap energy grows slightly from 1.27 to 1.31 eV for the single-phase samples with *x* = 0–0.15 according to the corresponding Tauc plots (Figure 3B). The second peak in energy is approximately equal to the electron transfer energy from slightly hybridized I *5p*–Sn *5p* orbitals localized below the top of the valence band to hybridized I *5p*–Sn *5s* orbitals of the conduction band.

As the indium concentration in the composition of the materials increases, the content of the CsInI_4_ phase increases, as can be seen from the XRD data, and this is manifested in the absorption spectrum of the sample *x* = 0.5 (~370 nm). This absorption edge is approximately equal to ~3.0 eV and relates to transitions in the electronic structure of the CsInI_4_ phase (Figure 3B), namely, the electron transition from the I *5p* orbitals (top valence band) to the hybridized I *5p*–In *5s* orbitals (conduction band bottom). The experimental *E*_*g*_ differs from the calculated bandgap value presented in Persson (2016).

**Figure 4:**
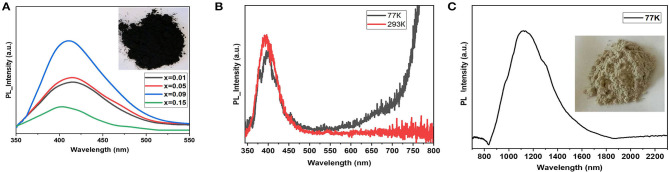
Photoluminescence emission spectra of **(A)** the samples Cs_2−x_Sn_1−x_In_*x*_I_6−2x_ (*x* = 0.01–0.15) at 293 K, and **(B)** and **(C)** PL emission of pure phase CsInI_4_ at 293 and 77 K in visible and infrared (IR) range, respectively.

Figure 4A shows photoluminescence spectra of the samples. The C_2_SnI_6_ perovskite phase demonstrates rather moderate intensity of luminescence with the 337-nm (3.68 eV) excitation laser, while its In-doped analog shows an intensive violet band at 410 ± 10 nm. Its intensity increases with an increase in the doping level *x*. Weaker broad maxima in 450–480 nm regions are observed for the samples with lower substitution rate.

Most likely, the PL of the solid solutions Cs_2−x_Sn_1−x_In_*x*_I_6−2x_ (*x* = [0; 0.1]) corresponds to cascade relaxation of electrons from the high free levels (anti-bonding derivative of Sn5p^0^ orbital) to the valence band (Xiao et al., 2015). It has been demonstrated that decrease of the temperature up to 77 K leads to devolution of the PL process. Such behavior is typical for materials with a gap slightly larger than the excitation laser wavelength. The visible photoluminescence in Cs_2_SnI_6_ phase is observed as a result of complex relaxation processes. The relaxation of the excited electron to the bottom of the conduction band occurs with visible luminescent process, and the following transition to the top of the valence band has a much lower energy than the observed PL process. Participation of deep levels originated from the point defects (*V*_*I*_) and is another possible reason for the PL effect in the visible diapason (Maughan et al., 2018).

For comparison, the gray cesium iodoindate(III) shows intensive PL bands at 380 ± 10 nm and 400 ± 10 nm in the visible region and intensive PL process in IR diapason at 1,170 ± 20 nm (1.16 eV) (Figures 4B,C). The 380-nm (3.26 eV) band correlates with the experimental band gap transition above while the second maximum in the visible range probably corresponds to shallow defects or self-trapped exciton processes. Intensive IR photoluminescence also originates from self-defect in cesium iodoindate, but the deep level is attributed to deficiency in iodine sublattice.

**Figure 5:**
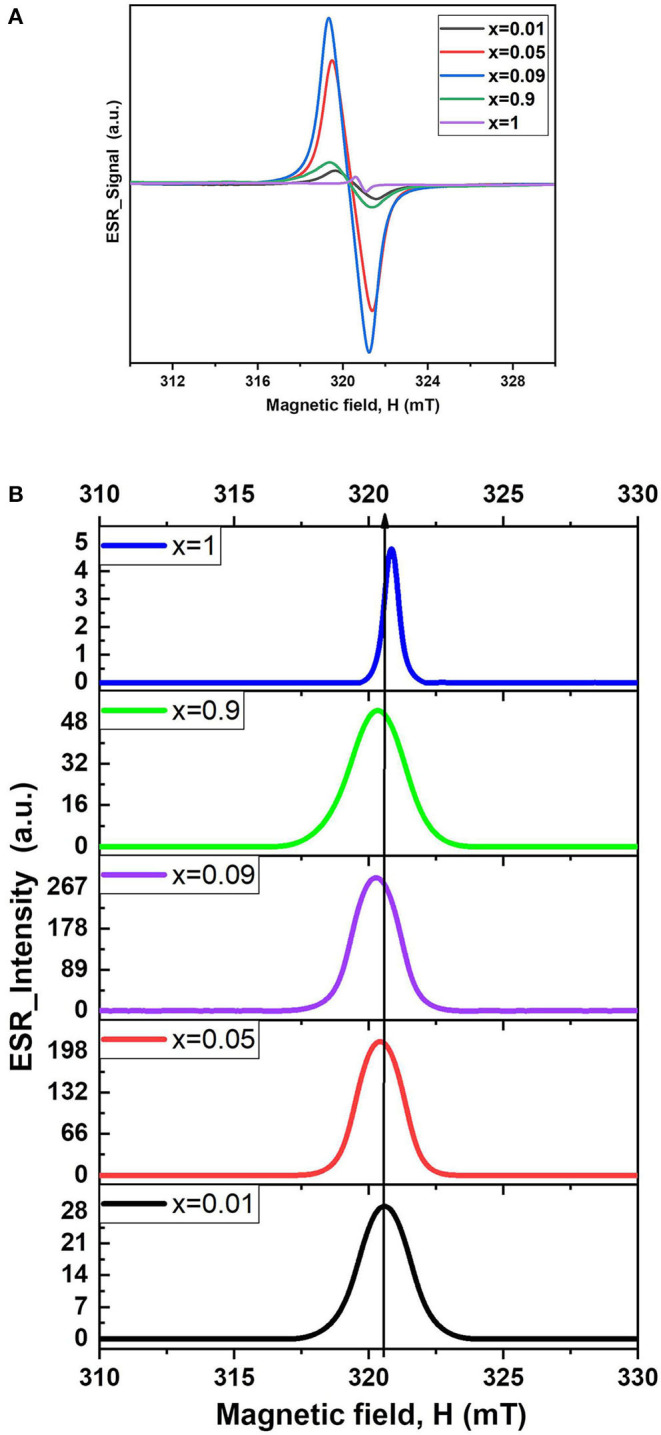
ESR spectra of Cs_2−x_Sn_1−x_In_*x*_I_6−2x_ (with different ratio of *x*) at 4.2 K **(A)** in derivative form and **(B)** in integrated form.

It is remarkable that the electron spin resonance effect is more significant for the cesium iodostannate(IV) phase with doped indium than for the pure phase. The ESR spectra for the Cs_2−x_Sn_1−x_In_*x*_I_6−2x_ (*x* = 0.01, 0.05, 0.09, 0.9, and 1) shown in Figure 5 demonstrate week shift of the resonance towards lower field values by the doping of indium from *x* = 0.01 to *x* = 0.9, resulting in the shift of the *g*-factor from 2.0045 to 2.0065. In the range of *x* = 0.01 to *x* = 0.09, the integral intensity of the ESR spectra grows up with increase of the substitution level *x* in the solid solution (Table 2), suggesting increment of the defects. The values of the *g*-factors and the areas calculated by the integration are given in Table 2. The spectra of the two-phase composition with *x* of 0.9 include less intensive and broad maximum at 321.2 mT. Probably, there could be an input shoulder related to the tin-doped cesium tetraiodoindate phase while the largest maximum corresponds to the spectrum of the solid solution Cs_2−x_Sn_1−x_In_*x*_I_6−2x_ saturated by In^3+^. Lower ESR resonance in CsInI_4_ phase, likely, attributed to lower defect concentration in cesium tetraiodoindate as a result of tetrahedral environment of indium and lower doping level.

**Table 2:** ESR spectra characteristics.

**Sample composition**	**Substitution rate *x***	**Integration curve area**	***g*-factor**
Cs_1.99_Sn_0.99_In_0.01_I_5.98_	0.01	68.6	2.0045
Cs_1.95_Sn_0.95_In_0.05_I_5.9_	0.05	440.4	2.0051
Cs_1.9_Sn_0.9_In_0.1_I_5.8_	0.09	604.9	2.0054
Cs_1.1_Sn_0.1_In_0.9_I_4.2_	0.9	134	2.0065
CsInI_4_	1	3.4	2.0029

## Conclusions

The perovskite-like phase Cs_2_SnI_6_ forms substitution solid solutions, changing tin to indium, forming a substitution solid solution of about 10 at.% of dopant. Increase of the indium percentage leads to growth of ESR and photoluminescence effects for the material. The successful experience in heterovalent substitution of tin(IV) with elementary indium and iodine as precursors opens new challenges for “improving” the characteristics of cesium iodostannate for its application in photovoltaics or optoelectronic devices. Weak degradation of doped phase in comparison to pure Cs_2_SnI_6_ is a strong advantage of the new investigated materials. Analysis of optical properties has shown an increase of absorption coefficient of the material that originated from growth of occupancy in the valence band. The intensity of blue photoluminescence also grows with a substitution rate up to 15%.

## Data Availability Statement

All datasets generated for this study are included in the article/supplementary material.

## Author Contributions

SU: synthesis of the samples, Raman spectroscopy experiments and discussion, optical spectroscopy and discussion, ESR experiments. AG: PL spectroscopy and ESR spectroscopy data discussion, correction of the text, and funding. LL: PL spectroscopy experiments. AK: XRD measurements and discussion. KN: ESR experiments and correction of the text. SO: ESR experiment discussion. AS: XRD discussion and correction of the text.

## Conflict of Interest

The authors declare that the research was conducted in the absence of any commercial or financial relationships that could be construed as a potential conflict of interest.
